# The first retinoblastoma local experience in Kuwait: A retrospective case series

**DOI:** 10.1016/j.ajoc.2023.101988

**Published:** 2023-12-29

**Authors:** Abdulaziz Alotaibi, Mohammad Karam, Allaa Roto, Sulaiman Alshuaib, Fatmah Alrabah, Alaa Alali

**Affiliations:** a AlBahar Ophthalmology Center, Ibn Sina Hospital, Ministry of Health, Kuwait; b Kuwait Board of Ophthalmology, Kuwait Institute for Medical Specializations (KIMS), Kuwait; c Dasman Diabetes Institute, Kuwait

## Abstract

**Background:**

To report the first series of retinoblastoma (RB) cases that were managed locally in Kuwait by the retinoblastoma team that was established during the COVID-19 pandemic.

**Results:**

Six cases with RB were included in this study. The ages ranged from 3 months to 2 years with a male to female ratio of 2:1. All cases presented with an abnormal pupillary reflex with or without strabismus. Examination findings mostly showed leukocoria and an intra-retinal mass with calcification with or without vitreous seeding. Most cases were unilateral except for one case, which had bilateral RB. International classification of RB staging ranged from group B to E. Multidisciplinary approach was followed to manage these cases by applying a well-set protocol created by the RB team. Each case was treated according to grade at presentation.

**Conclusion:**

COVID-19 pandemic revolutionized the standard of care for RB in Kuwait and mandated the establishment of a multidisciplinary team to follow a standardized protocol to manage RB cases successfully.

## 1 Introduction

Retinoblastoma (RB) is the most common intraocular malignant tumour in children caused by a mutation in both alleles of the RB1 tumour suppressor gene.^1^ Its worldwide incidence is 1 in every 16,000 to 18,000 live births. The Global Retinoblastoma Study Group investigated 4351 patients from 153 countries reveals a global disparity, with 84.7 % from low- and middle-income countries, showcasing distinct patterns in diagnosis age, disease presentation, and metastasis rates.^2^ Highlighting the disparities in retinoblastoma outcomes, another study of 4064 children from 149 countries underscores the stark contrast in survival rates, with a 3-year survival rate of 99.5 % for high-income countries, 57.3 % for low-income countries, and identifies residence in low-income countries, advanced tumor stage, and older age at diagnosis as independent factors associated with worse survival.^3^ The primary goal of management is to save the patient's life, whereas secondary goals include saving the eye and maintaining good visual outcomes.^1^

Prior to COVID-19 pandemic, patients diagnosed with RB in Kuwait were sent abroad for treatment. However, due to travel restrictions during the pandemic, the first retinoblastoma team was established to manage RB cases locally. The current study reports a series of RB cases to reflect the local experience with the management of RB.

### 1.1 Case presentations

**Fig. 1:**
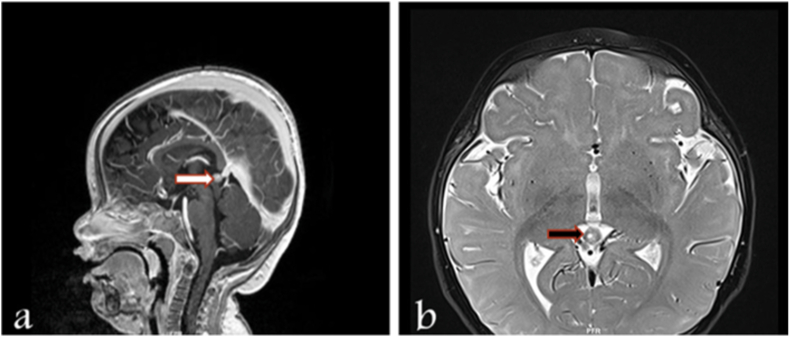
**MRI brain findings of case 2 demonstrating pineal involvement (a) sagittal plane (b) axial plane.** The pineal gland was relatively prominent (arrow) with heterogeneous appearance and enhancement. No sellar or suprasellar mass was noted.

**Fig. 2:**
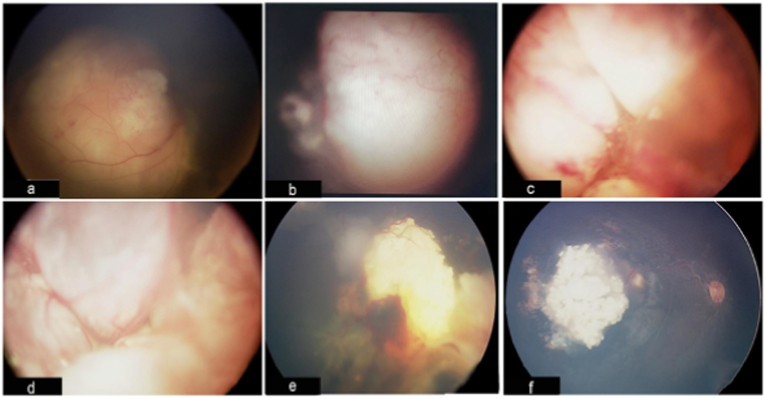
**Retcam images of posterior segment of the retinoblastoma cases at presentation: (a) Case 1 OD -** large single subretinal mass obscuring the temporal border of the optic nerve with calcification and small cuff of SRF without vitreous. **(b) Case 2 OS -** advanced group D RB obscuring the optic nerve with multiple vitreous seeds**. (c) Case 3 OS -** large vascular retrolental mass obscuring the optic disc with anterior vitreous seeding. **(d) Case 4 OS -** retro-lental mass obscuring the view to examine the optic nerve and macula. **(e) Case 5 OS -** exudative RD with pre retinal haemorrhages and vitreous seeds**. (f) Case 6 OD** - inactive chronic calcified solitary lesion without seeding. Key: OD: Right Eye; OS: Left Eye.

A total of six patients with RB presented between from February 2020 and December 2021 were identified with a median (range) age of 1 year (3 months-4 years). Their main presentation was leukocoria noticed by parents (Table 1). Four patients were managed locally, while two cases were followed up locally after their initial management abroad prior to the pandemic. For all newly diagnosed cases, urgent EUA and MRI brain and orbit were performed within the first 48 hours of presentation. A single case showed pineal gland involvement (Fig. 1). Case 5 is a familial case and was treated abroad by means of enucleation. Case 4 is a 12-month-old child who was diagnosed with group E retinoblastoma and accordingly had left eye enucleation. Moreover, Retcam images demonstrated various presentations of retinoblastoma cases, ranging from large subretinal masses with calcification to advanced cases with vitreous seeds and vascular retrolental masses (Fig. 2).

**Table 1:** **Baseline characteristics and management of included cases.** Key: IAC: Intra-arterial chemotherapy; * systemic chemotherapy included carboplatin, etoposide, and vincristine.

Case	Age at Presentation	Sex	Presentation	Laterality	Group at presentation	Treatment	Cycles of Chemotherapy	Enucleation and prosthesis
1	4 months	M	Right leukocoria	Unilateral	B	Initally: Pre chemo cryotherapy, systemic chemotherapy Follow up EUAs showed progression of the tumor activity thus enucleation was performed	6 cycles*	Yes
2	8 months	F	Left leukocoria with strabismus	Unilateral with pineal gland involvement	D (obsucuring the optic nerve)	Systemic chemotherapy and unilateral enucleation	6 cycles*	Yes
3	2 years	F	Left leukocoria with strabismus	Unilateral	E	Enucleation	N/A	Yes
4	1 year	M	Left leukocoria	Unilateral	E	Enucleation	N/A	Yes
5	Initially diagnosed and managed abroad at 1.5 years of age and followed up at 3 years of age	M	Follow up	Bilateral	Right: inactive well treated tumor Left: active stage D	Abroad: Laser therapy x3 (OU), IAC x3 (OU) OS cryotherapy and brachytherapy Local: Systemic chemotherapy x6, and unilateral enucleation (OS)	6 cycles*	Yes
6	Initially diagnosed and managed abroad at 2 years of age and followed up at 4 years of age	M	Follow up	Unilateral	Right: inactive well treated tumor	Abroad: IAC and laser therapy Local: Routine EUA's with no active intervention required	N/A	No

The RB international classification of the cases ranged from B to E (Table 1). Regarding management outcomes, all six cases have survived with no extra-ocular metastasis and one eye was salvaged without the need for enucleation. The average follow-up time for the cases was 3–6 months post-intervention. Despite all precautions taken to facilitate managing suspected RB cases, five cases ended up with enucleation as most cases were advanced group D and E (Table 1). These cases sought medical assessment for the first time with advanced stage and associated strabismus.

**Fig. 3:**
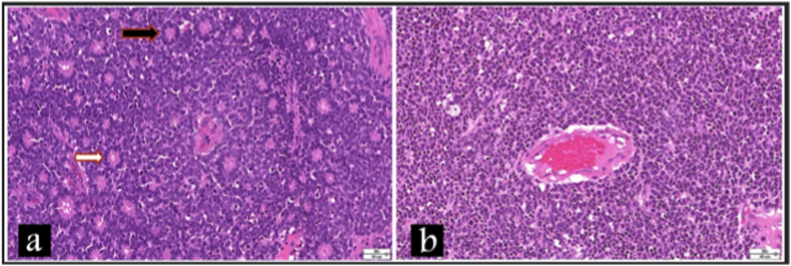
**Pathology photomicrographs of the enucleated eyes of retinoblastoma cases: (a) Left eye of case 4 –** it shows grade 2 retinoblastoma composed of sheets of malignant small round blue cell admixed with Flexner-Wintersteiner rosettes (white arrow) and Homer Wright rosettes (black arrow) (H&E stain, x200). Which is similar to case 1 and case 5. **(b) Left eye of case 3 –** it shows poorly differentiated retinoblastoma composed of malignant small round blue cell exhibiting scant cytoplasm, hyperchromatic nuclei with perivascular cuffing (H&E stain; x200). (For interpretation of the references to colour in this figure legend, the reader is referred to the Web version of this article.)

Pathology photomicrographs of the enucleated eye of case 4 demonstrated grade 2 RB with Flexner-Wintersteiner rosettes and Homer Wright rosettes that is similar in appearance to case 1 and case 5 (Fig. 3a). On the other hand, the pathology of the left eye of case 3 revealed a poorly differentiated RB (Fig. 3b).

## 2 Discussion

In this case series, a total of 6 cases of RB were reviewed with the initial parents’ alarming observation of abnormal pupillary reflex (Table 2). Three out of six cases (50 %) had associated strabismus.^4^ This is consistent with a systematic review by Mattosinho et al. that reported leukocoria and strabismus to be the most common presenting symptoms of RB.^5^ The ages of the included cases ranged from 3 months to 2 years, which is the typical age presentation for RB.^5^

**Table 2:** Clinical and radiological features of the included cases. Key: OD: Right Eye; OS: Left Eye: OU: Both Eyes; AS: Anterior Segment; PS: Posterior Segment; AC: Anterior Chamber; D&Q: Deep and Quiet; IOP: Intraocular pressure; NVI: Neovascularisation of the iris. SRF: subretinal fluid.

Case	Presenting Symptom	Family history	Initial EUA at presentation		Initial MRI Findings
			OD	OS	
1	White pupillary reflex OD for 4 days	No	Leukocoria AS: normal with no NVI IOP: 19 mmHg PS: large single subretinal mass obscuring the temporal border of the optic nerve with calcification and small cuff of SRF without vitreous seeds.	AS: normal with no NVI IOP: 16 mmHg PS: normal Scleral depression: normal	Solid right ocular lesion (12x9x8 mm) involving temporal retina with endophytic growth covering the optic disc
2	White pupillary reflex OS for 2 weeks	No	AS: normal with no NVI IOP: 14 mmHg PS: normal Scleral depression: normal	Leukocoria AS: normal with no NVI IOP: 19 mmHg PS: advanced group D RB obscuring the optic nerve with multiple vitreous seeds	Large left RB with suspected optic disc and pineal gland involvement
3	White pupillary reflex OS for 1 month and outward deviation OS for 1 week	No	AS: normal with no NVI IOP: 21 mmHg PS: normal Scleral depression: normal	Leukocoria AS: NVI IOP: 27 mmHg PS: large vascular retrolental mass obscuring the optic disc with anterior vitreous seeding	Soft tissue mass with left optic nerve enhancement
4	White pupillary reflex OS for 2-months	No	AS: normal with no NVI IOP: normal PS: normal Scleral depression: normal	Leukocoria AS: NVI IOP: 27 PS: retro-lental mass obscuring the view of the optic nerve and macula	Left intraocular mass lesion without optic nerve invasion with retinal detachment
5	White pupillary reflex and inward deviation OS as a follow-up from being managed abroad from presentation	Yes	AS: normal IOP: 21 mmHg PS: inactive grape-shaped calcified tumor	Leukocoria OS AS: normal with no NVI IOP: 8 mmHg PS: exudative RD with pre retinal hemorrhages and vitreous seeds	Left eye retinal detachment with nodular lesion noted near the left optic disc worrisome for tumor recurrence and normally appearing optic nerve. The right eye showed no active tumors with old calcifications.
6	White pupillary reflex and inward deviation OD for 4 months as a follow-up	No	Leukocoria AS: normal IOP: 18 mmHg PS: inactive chronic calcified solitary lesion without seeding	AS: normal with no NVI IOP: 17 mmHg PS: normal Scleral depression: normal	Right enhancing retinal lesion with calcification. Optic nerve-sheath complexes and extraocular muscles show normal features bilaterally.

Most cases presented to the emergency department rather than an outpatient clinic. The detection of RB by first on-call residents and the subsequent step-wise management of suspected cases by urgent admission, MRI imaging, and EUA by a pediatric retina specialist reflects the high level of preparation of ophthalmology emergency services. This was achieved because of a well structured RB protocol that was created by the multidisciplinary retinoblastoma team. This team involved specialists in pediatric ophthalmology, vitreoretinal surgery, oculoplasty, pediatric neuroradiology, pediatric medical oncology, radiation oncology, social worker, pathology, and clinical genetics. The RB protocol aimed to emphasize the importance of time in managing RB and prevent any delay that can negatively affect the overall outcome by providing all facilitations needed for urgent admission and direct contact with RB team members.

The timing of RB diagnosis and urgent referral to a specialized center is one of the most important prognostic factors in terms of globe salvage and survival.^6^ Kaliki et al., in 2021 compared low-, middle- and high-income countries regarding the adverse effects of the more advanced stage of the tumor with poorer outcomes and survival rates with increased lag time between RB presentation and management.^6^ They reported that the median lag time in low- and middle-income countries is 69 days, whereas the high-income countries including the UK and USA had medians of 31 and 45 days respectively.^6^ In the current study, the time from presentation to diagnosis and management ranged from 24 hours to one week (median of 4 days), which was comparable to the high-income countries’ readiness to deliver the management promptly.

Having a clear RB protocol to follow by all ophthalmologists was one of the key goals of the established RB team and the primary factor behind the good results of such a first-time experience locally. This protocol would be beneficial for any ophthalmic care institute willing to prepare an effective and timely fashioned management for such emergency.

The global guidelines were followed in terms of adjuvant, neo-adjuvant chemotherapy, surgery as well as follow-up strategies (Table 1).^7^ Although there was a current shift in RB classification to the TNM staging, the RB international classification was followed in managing these cases for simplicity, matching the new experience of the first local RB team.^8^ The pandemic travel restriction protocols lead to a delay in diagnosis of RB causing loss of lives worldwide.^9^ Bansal et al. investigated the effect of lockdown nationwide of RB cases in India with 2 % childhood mortality due to extra-cranial extension.^4^

The enucleation in most cases, due to the detection of advanced group D and E, reflects the lack of parents' awareness regarding warning signs of a child's eye development. Naser et al. investigated the RB knowledge and awareness in around 3600 participants from Jordan, Iraq, and KSA demonstrating a relatively low knowledge score (6.25 out of 21).^10^ This highlights the importance of raising awareness amongst parents regarding RB presenting signs as well as providing genetic counselling for familial cases.

## 3 Conclusions

COVID-19 pandemic revolutionized the standard of care for RB and allowed the creation of a multidisciplinary team. In the current case series, six cases of RB were successfully managed and followed up during COVID-19 pandemic. This study featured the importance of the preparation of the health care system to deliver a high quality of care under global emergencies such as COVID-19 pandemic. Moreover, the delayed presentation of most cases highlights the importance of raising awareness regarding signs of retinoblastoma amongst parents, which could be achieved through government sponsored campaigns. Further follow-up of these cases is required to study the survival rate and fully evaluate the effectiveness of the local treatment protocol.

## Funding

The author(s) received no financial support for the research, authorship, and/or publication of this article.

## Patient consent

Consent to publish this case report has been obtained from the patients and this report does not contain any personal identifying information.

## CRediT authorship contribution statement

**Abdulaziz Alotaibi:** Conceptualization, Data curation, Formal analysis, Software, Validation, Writing – original draft, Writing – review & editing. **Mohammad Karam:** Conceptualization, Formal analysis, Resources, Validation, Visualization, Writing – original draft, Writing – review & editing. **Allaa Roto:** Methodology, Writing – original draft. **Sulaiman Alshuaib:** Investigation, Writing – original draft, Writing – review & editing. **Fatmah Alrabah:** Software, Supervision. **Alaa Alali:** Formal analysis, Project administration, Resources, Supervision, Writing – review & editing.

## Declaration of competing interest

The authors declare that they have no known competing financial interests or personal relationships that could have appeared to influence the work reported in this paper.
